# A UPLC-MS/MS method for simultaneous quantification of pairs of oleanene- and ursane-type triterpenoid saponins and their major metabolites in mice plasma and its application to a comparative pharmacokinetic study

**DOI:** 10.1039/c8ra00739j

**Published:** 2018-02-26

**Authors:** Wenwen Zhao, Zongyang Liu, Weiwei Guo, Kui Luo, Jie Yang, Wei Gao, Xia Wu, Xiaoqing Chen

**Affiliations:** School of Traditional Chinese Medicine, Beijing Key Lab of TCM Collateral Disease Theory Research, Capital Medical University Beijing 100069 China wuxia6710@163.com cxqcpu@163.com; Core Facilities Center, Capital Medical University Beijing 100069 China; Department of Chinese Medicines Analysis, China Pharmaceutical University Nanjing 210009 China; Beijing Key Lab of TCM Collateral Disease Theory Research, Capital Medical University Beijing 100069 China

## Abstract

Ilexhainanoside D (IhD) and Ilexsaponin A_1_ (IsA) are a pair of oleanene- and ursane-type triterpenoid saponins, which are also the main bioactive pharmaceutical ingredients of *Ilex hainanensis* Merr. with great potential to treat non-alcoholic fatty liver disease (NAFLD). The pharmacokinetics of four representative triterpenoids in mice were investigated in this study, which were IhD, IsA and their major metabolites 3β, 19α-dihydroxyolean-12-ene-24, 28-dioic acid (ID) and Ilexgenin A (IA). A sensitive and accurate UPLC-MS/MS method was developed and validated for the simultaneous quantitative determination of IhD, IsA, ID and IA in control and NAFLD mice plasma after oral administration of the total saponins of *I. hainanensis* (the contents of IhD and IsA were 41.6% and 54.4%, respectively). The results revealed that the pharmacokinetic behaviors could be changed in NAFLD mice compared with control mice. The area under the plasma drug concentration–time curve and maximum plasma concentrations of IhD and IsA were greatly decreased in the NAFLD mice. However, the main residence time of ID and IA were greatly increased in the NAFLD mice. The results revealed that this method could be used to analyze two pairs of triterpenoid isomers in biological samples.

## Abstract 2

Pharmacokinetic study of pairs of oleanene- and ursane-type triterpenoid saponins and their major metabolites in control & NAFLD mice.

## Graphical abstract

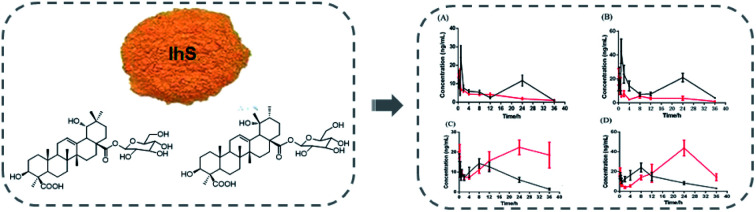

## 1. Introduction

**Fig. 1:**
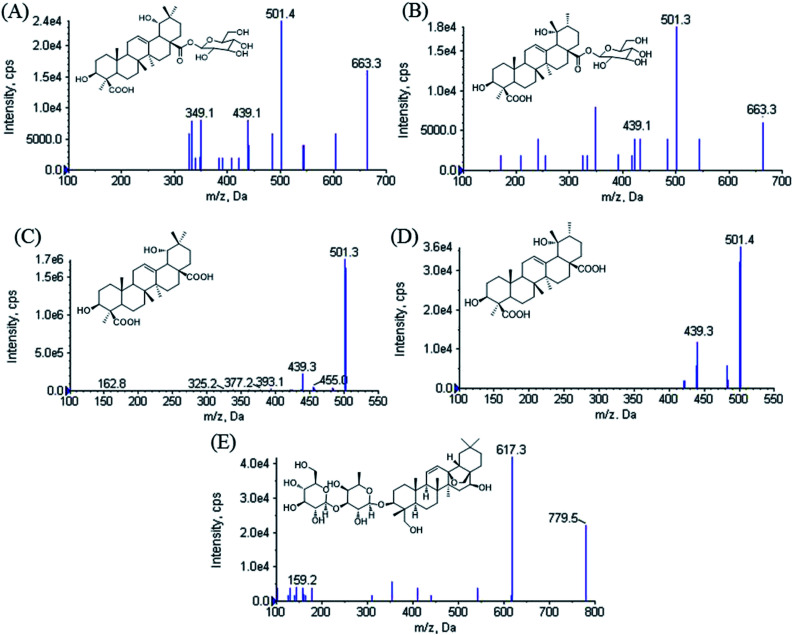
Product ion scan spectra of [M − H]^−^ for (A) ilexhainanoside D, (B) ilexsaponin A_1_, (C) 3β, 19α-dihydroxyolean-12-ene-24, 28-dioic acid, (D) ilexgenin A and (E) saikosaponin A.

Ilexhainanoside D (IhD), Ilexsaponin A_1_ (IsA), 3β, 19α-dihydroxyolean-12-ene-24, 28-dioic acid (ID) and Ilexgenin A (IA) are two pairs of oleanene- and ursane-type triterpenoids (structures shown in Fig. 1). They are the major triterpenoid glycosides and aglycones in the leaves of *Ilex hainanensis* Merr. (Aquifoliaceae), named Shan Lv-Cha in Chinese.^1^ This herbal medicine is commonly used as a kind of tea product as well as a folk medicine for treating hypertension, dyslipidemia and inflammation.^1^ It has been demonstrated that the extracts from *I. hainanensis* could reduce the levels of blood lipids and improve diet-induced non-alcoholic fatty liver disease (NAFLD).^2^ Various kinds of preparations containing *I. hainanensis* extract are available in clinics, such as Shanlvcha Jiangya Tablets and Shanlvcha Jiangya Capsules.^3^

Phytochemical studies revealed that main components of *I. hainanensis* were triterpene glycosides, triterpenes and flavonoids.^4^ IhD and IsA, ID and IA are the major bioactive constituents in *I. hainanensis,* which show great pharmacological effects, such as cardiovascular and cerebrovascular protection activities in mice^5^ and protective effects against NAFLD in rats.^6^ IhD and IsA usually exist in pairs in the plants, and their contents are 0.5–1% and 1–4%, respectively.^7^

Till now, high-performance liquid chromatography with ultraviolet detection (HPLC-UV)^8^ and high-performance liquid chromatography coupled with tandem mass spectrometry (HPLC-MS)^9^ methods have been reported to quantify IsA and IA in biological samples. However, the pharmacokinetic (PK) study after oral administration of IsA or IhD is still very limited. As the major metabolites of IhD and IsA,^10^ ID and IA also exist in *I. hainanensis*, so the determination of the aglycones is controversial since they can be directly absorbed from the plant extract and also can be transformed from glycosides *in vivo*.

NAFLD is the manifestation of the metabolic syndrome in liver, which is characterized by an excessively high accumulation of fat deposits in the liver resulting from causes other than chronic alcohol abuse.^11^ It is a risk factor for a variety of metabolic diseases, including obesity, type-2 diabetes, and dyslipidaemia.^12,13^ However, to date, the mechanisms underlying NAFLD pathogenesis remain unclear. It is essential to search for high-effective agents to ameliorate NAFLD. C57BL/6J mice are susceptible to metabolic disease, and the mouse model of NAFLD induced by high-fat diet is also safe, reliable and easily repeatable since the pathogenesis is similar to the human body.^14^ Researches have shown that metabolism of the drugs can be influenced in NAFLD mice.^15^ As a result, it is meaningful to research on the PK behaviors of the triterpenoid isomers in mice, which are investigated as promising candidates for the treatment of NAFLD.

In order to investigate the differences in the metabolic characteristics of the isomers, the methods of biosample preparation and chromatographic condition were evaluated to develop a sensitive and accurate UPLC-ESI-MS/MS method to simultaneously detect IsA, IhD, IA and ID in mice plasma. The method was applied to a pharmacokinetic study in mice after oral administration of the total saponins of *I. hainanensis* (IhS). The differences of the metabolism profiles of the two pairs of the isomers and the PK behaviors in control and NAFLD mice were investigated. The obtained results would be helpful for evaluating the clinical applications of this herb medicine.

## 2. Experimental

### 2.1. Chemicals, material and reagents

Saikosaponin A (internal standard, IS) was obtained from National Institute for the Control of Pharmaceutical and Biological Products (Beijing, China, 110777-201510). IhD, IsA, ID and IA were isolated from *I. hainanensis* in our previous studies.^16–18^ Their structures were showed in Fig. 1 and the purity of all chemicals were determined to be more than 98% by HPLC.

The leaves of *I. hainanensis* were collected from Guangxi province of China in October 2015 and were identified and authenticated by Ke Zan (National Institutes for Food and Drug Control, Beijing, China). A voucher specimen (no. 20151101) was deposited at School of Traditional Chinese Medicine, Capital Medical University, China.

HPLC-grade ammonium formate, formic acid and ethyl acetate were purchased from Thermo Fisher Scientific (Hudson, NH, USA). Methanol (LC-MS grade) was purchased from Thermo Fisher Scientific (Hudson, NH, USA). Deionized water prepared by the Millipore system (Molsheim, France) was used for all the preparations. Column chromatography was performed on Macroporous resin D101 (Tianjin Haiguang Chemical Co., Ltd., Tianjin, P. R. China), polyamide (30–60, Shanghai Aladdin Industrial Co., Ltd., Shanghai, P. R. China) and Sephadex LH-20 (Pharmacia Fine Chemical Co., Ltd., Germany). Other reagents were of analytical grade.

### 2.2. Equipment and LC/MS condition

The UPLC-MS/MS system consisted of an Agilent 1290 series LC system (Agilent, Mississauga, Ontario, Canada) connected to an AB Sciex QTrap® 6500 hybrid linear ion-trap triple quadrupole mass spectrometer equipped with a Turbo Spray source (AB Sciex, Concord, Ontario, Canada). The mass spectrometer was operated in negative ionization mode and the data were acquired using the Analyst 1.6.1 software on a Microsoft Windows XP Professional operating platform.

Chromatographic separation was achieved using a Zorbax SB-C18 column (2.1 mm × 100 mm, 1.8 μm, Agilent Technologies, Wilmington, DE, USA) maintained at 30 °C. The mobile phase comprising of solvent A (0.1% formic acid in 5 mM ammonium formate) and solvent B (methanol) was pumped isocratically in the ratio of 25 : 75, v/v at a flow rate of 0.3 mL min^−1^. All solvents were passed through 0.22 μm membrane filter (Millipore, USA) and degassed ultrasonically (Kun Shan Ultrasonic Instruments Co., Ltd, Jiangsu, P. R. China) for 20 min prior to use. The injection volume was 5 μL and the time taken for each analytical run was 6 min. The sampling needle was washed with mobile phase between each injection.

Mass spectrometric parameters were optimized *via* direct infusion of standard solutions. The spray voltage was maintained at −4500 V. The ion spray temperature was optimized at 555 °C. Collisionally activated dissociation (CAD) gas was set at medium level. Curtain gas and ion source gases 1 and 2 were supplied at 35, 55 and 55 psi, respectively.

All analytes were monitored *via* multiple reaction monitoring (MRM) at *m*/*z* 663.5 → 501.5 for IhD and IsA, *m*/*z* 501.2 → 439.2 for ID and IA, *m*/*z* 779.2 → 617.5 for IS, respectively. Declustering potentials (DP) for IhD, IsA, ID, IA and IS were set at −180 V. The collision energies (CE) were −40, −40, −45, −45 and −48 V, respectively. The mobile phase flow was diverted to the waste before 0.5 min and after 5.5 min during the chromatographic run to protect the mass spectrometer from contamination and reduce the solvent load in the source.

### 2.3. Preparation of the total saponins of *I. hainanensis*

The dried leaves of *I. hainanensis* (5 kg) were extracted with 75% ethanol for 2 times and 1 h per time. The extraction solutions were combined and evaporated in vacuum. And then the extract was fractioned and subjected to a D101 macroporous resin column and eluted with a gradient of EtOH/H_2_O (0 : 100, 30 : 70, 50 : 50 and 70 : 30, v/v). The 70% EtOH fraction was separated by column chromatography on Sephadex LH-20 (MeOH) to clear away phenols. IhS was then prepared after purification by semi-preparative HPLC (ACN/H_2_O, 85 : 15, v/v) to make sure there were not ID and IA in them. The contents of IhD and IsA were 41.6% and 54.4%, respectively. ID and IA were not detected by HPLC.

### 2.4. Preparation of standard solutions, calibration samples and quality control samples

The stock solutions (1 mg mL^−1^) of IhD, IsA, ID, IA and IS were prepared in methanol. Stock solutions of IhD, IsA, ID and IA were then mixed and serially diluted with methanol to obtain standard solutions containing 8–3200 ng mL^−1^ IhD and IsA and 160–3200 ng mL^−1^ ID and IA. The IS working solution of 500 ng mL^−1^ was also prepared by dilution of the stock solution with methanol. All solutions were stored at 4 °C and brought to room temperature before use.

Mixed calibration standards containing IhD and IsA 0.5, 2.5, 5, 10, 50, 100, 200 ng mL^−1^, ID and IA 10, 20, 30, 50, 75, 100, 200 ng mL^−1^ were prepared by spiking aliquots of the standard solutions into blank plasma (1 : 16). The low, middle and high quality control (QC) samples containing IhD and IsA 1, 50 and 180 ng mL^−1^, ID and IA 20, 50 and 180 ng mL^−1^ were prepared independently in the same fashion.

### 2.5. Biosample preparation

The frozen plasma samples were thawed at room temperature and vortexed thoroughly before analysis. To an aliquot of 50 μL plasma sample, 10 μL of IS solution (500 ng mL^−1^), 10 μL of methanol and 200 μL of 0.2% formic acid were added and vortex-mixing for 30 s. Liquid–liquid extraction (LLE) was carried out with 1 mL ethyl acetate and vortexed for 5 min. After centrifugation at 15 000 rpm for 5 min, the supernatant was transferred to another vial and evaporated to dryness at 37 °C under a gentle stream of nitrogen. The residue was reconstituted with 80 μL methanol/water (75 : 25, v/v), vortex-mixed for 1 min, and centrifuged at 15 000 rpm for 5 min. 5 μL of the supernatant was injected into the UPLC-MS/MS system for analysis.

### 2.6. Method validation

The specificity of the method was evaluated by comparing the chromatograms of blank plasma samples collected from six different mice with the corresponding spiked plasma samples at the lower limit of quantification (LLOQ). The areas of co-eluting interference peaks in blank plasma samples should be <20% of the peak areas of the analytes in LLOQ samples.

The calibration curves were constructed by establishing a linear regression function after 1/*x*^2^ weighting of the analyte/IS peak area ratio *versus* analyte concentration relationship. The acceptance criterion for a calibration curve was a correlation coefficient (*r*) > 0.995.

The precision and accuracy of this analytical method were evaluated using QC samples. For intra-day precision and accuracy, six replicates were analyzed at each concentration level. The inter-day precision and accuracy were determined by analyzing six replicates at each concentration level on three consecutive days. The accuracy was calculated as mean percent deviation (RE) of the observed concentration (*C*_obs_) from the spiked concentration (*C*_spi_), accuracy (% RE) = [(*C*_obs_ − *C*_spi_)/*C*_spi_] × 100. The precision was expressed by the relative standard deviation (RSD), precision (% RSD) = [standard deviation (SD)/mean *C*_obs_] × 100. The acceptable intra-day and inter-day precision is required to be less than 15% and the acceptable accuracy was required to be within 15% for all QC samples.

The recoveries of each analyte at three QC levels (*n* = 6) were determined by comparing the responses of the analytes from QC samples with the responses of analytes spiked in post-extracted samples at equivalent concentrations. Percent RSD of ±15% or better was the acceptable limit for all tested concentrations.

The matrix effect was evaluated by comparing the responses of analytes added into pre extracted plasma from untreated mice (A), with those of analytes dissolved in matrix component-free reconstitution solvent (B). When the ratios (A/B) 100 × of the analytes were between 85% and 115%, the matrix effect might be considered as negligible.

The stability of IhD and IsA with their metabolites ID and IA was investigated under different storage and processing conditions. The long-term stability at −20 °C was evaluated for 30 days. The short-term room temperature stability was investigated at room temperature for 4 h. The freeze–thaw stability was assessed by three consecutive freeze (−20 °C)–thaw (RT) cycles. Stability in processed samples in autosampler vials at 4 °C for 12 h was also assessed. The analytes were considered stable when the accuracy bias was within ±15% of the nominal concentrations.

### 2.7. Applications in pharmacokinetics studies

The developed UPLC-MS/MS method was applied to the pharmacokinetic studies of IhD, IsA, ID and IA after oral administration of IhS in the control and NAFLD mice. Male C57BL/6J mice (SPF) weighing 22–26 g were purchased from Vital River Laboratories (VRL) (Beijing, China). The experimental procedures were carried out following the guidelines of the Experimental Animal Care and Use Committee at the Capital Medical University (Beijing, China). All studies were approved by the Capital Medical University Animal Experiments and Experimental Animals Management Committee (IACUC Protocol no. AEEI-2016-023). Four mice were placed in one cage, and maintained under controlled room temperature (25 ± 2 °C) and humidity (60–70%) with day/night cycle (12 h/12 h). All animals had free access to food and water.

After acclimatization for 5 days, all mice were divided into the control group (*n* = 24) and the NAFLD group (*n* = 24) randomly. The control group was given with control diet and the NAFLD group was given with high-fat diet, in which 60% kcal% fat was added into the control diet.

After 4 weeks, all mice were fasted overnight for 12 h, and general biochemical parameters were evaluated, including triglyceride (TG), total cholesterol (TC), low density lipoprotein-cholesterol (LDL-c), high density lipoprotein-cholesterol (HDL-c), alanine aminotransferase (ALT), and aspartate aminotransferase (AST) in plasma. Six mice in each group were sacrificed randomly, the livers were collected and pathological changes in the liver tissues were observed by H&E staining to make sure the success of the NAFLD model. The concentrations of TG, TC, HDL-c, LDL-c, ALT, AST in plasma were determined using commercial kits (JianCheng Bioengineering Institute, Nanjing, China).

After continue feeding three days as before, both the control and NAFLD group were orally administered with IhS at 250 mg kg^−1^ (104 mg kg^−1^ IhD and 136 mg kg^−1^ IsA), which was suspended in 0.5% carboxymethyl cellulose sodium aqueous solution.

Blood samples were collected in heparinized tubes at 0.083, 0.25, 0.5, 0.75, 1, 2, 4, 8, 12, 24, 36 h after a single oral administration. The samples were immediately centrifuged at 3500 rpm for 10 min. The plasma was finally obtained and stored at −20 °C until analysis.

Pharmacokinetic parameters including half-life (*t*_1/2_), the area under the concentration–time curve (AUC), maximum plasma concentration (*C*_max_) and time (*T*_max_), mean residence time (MRT), the volume of distribution (*V*_d_) and plasma clearance (CL) were estimated by a non-compartmental method using Phoenix WinNonlin 7.0 (Certara, Princeton, NJ 08540 USA).

## 3. Results and discussion

### 3.1. Sample preparation and IS

**Fig. 2:**
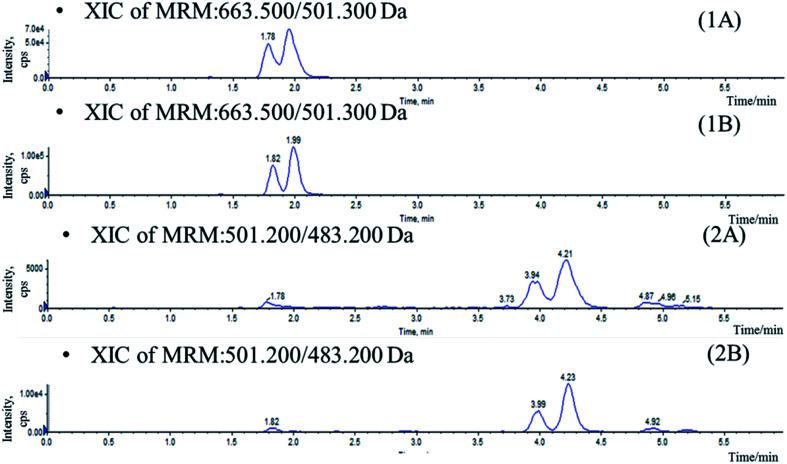
Chromatograms of blank plasma sample spiked with IhD, IsA, ID and IA prepared by protein precipitation (A) and LLE (B). (1) IhD and IsA, (2) ID and IA.

In the current study, attempts were made using protein precipitation and LLE^19^ which were simple and most widely used economical procedures than solid phase extraction (SPE). Protein precipitation with acetonitrile and methanol conduced to high background noise which affects the resolution of the isomers (Fig. 2). As a result, LLE was selected in which several organic extractants and the addition of acidifying/basifying agents were investigated for optimal extraction of analytes from biomatrix. Ethyl acetate appeared to be the best choice, yielding a higher extraction ratio and lower background interference. Moreover, the addition of 200 μL 0.2% formic acid to 50 μL plasma before extraction was found to increase recovery for the analytes. Saikosaponin A was used as IS owing to its similar characteristics to the four compounds during the sample preparation and analysis.

### 3.2. LC-MS optimization

Mass spectrometer parameters were optimized using a syringe pump (100.0 ng mL^−1^ in methanol for the analytes and IS). ESI sources with positive or negative ionization were tested for the determination. The results revealed that the analytes and IS were more sensitive in negative ionization mode. The ionspray voltage, the turbo spray temperature, the entrance potential (EP), especially the DP and CE for each transition were also optimized. The final parameters were shown in the Section 2.2.

**Fig. 3:**
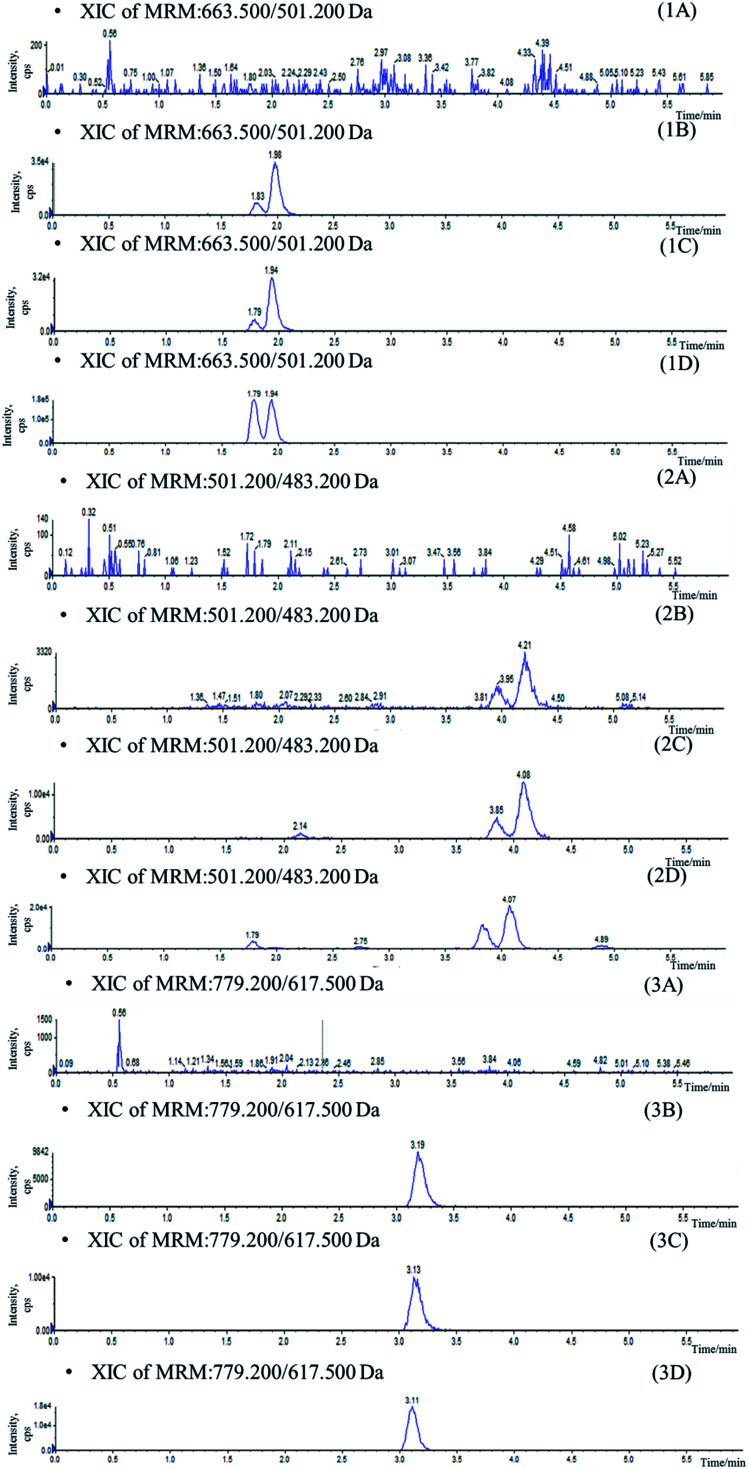
Typical chromatograms of blank plasma (A), blank plasma sample spiked with IhD, IsA, ID and IA at LLOQ and IS (B), plasma sample collected at 36 h after oral administration of IhS (C) and reference substance (D). (1) IhD and IsA, (2) ID and IA (3) IS.

Stationary phase and composition of mobile phase were investigated to obtain good chromatographic conditions. An Agilent Zorbax SB-C18 column (2.1 mm × 100 mm, 1.8 μm) was chosen in the present study for its good peak symmetry. Different mobile phases (acetonitrile–water and methanol–water or with different concentration of formic acid and ammonium formate) were examined to obtain efficient chromatography and relatively short run time for the analytes and IS. It was found that the methanol–water system was better than the acetonitrile–water system for its great resolution of the isomers, and the addition of ammonium formate in mobile phases could increase the ionization of the analytes and IS. The retention times for IhD, IsA, ID, IA and IS were 1.7, 1.9, 3.6, 3.8 and 2.9 min, respectively (Fig. 3).

### 3.3. Method validation

Specificity was assessed by comparing the chromatograms of six different batches of blank mice plasma with the corresponding spiked plasma. Under the optimized LC-MS conditions, the representative chromatograms of blank plasma, blank plasma spiked with the analytes, and plasma of samples are shown in Fig. 3. No significant interferences from endogenous substances were observed at the retention times of the analytes and IS, suggesting good selectivity of the developed method.

The matrix effect of analytes must be investigated during the analysis since the ionization of the analytes may be influenced by the co-elute, undetected endogenous matrix compounds. In this assay, it was demonstrated that no signal suppression or enhancement were found under these conditions (Table 1).

**Table 1:** Matrix effects and the extraction recoveries of the four analytes in mice plasma (*n* = 6)

Analytes	Spiked-concentration (ng mL^−1^)	Matrix effects (%)	RSD (%)	Recovery (%)	RSD (%)
Ilexhainanoside D	1	101.10	4.38	92.88	7.18
	50	103.42	8.59	80.20	4.43
	160	97.59	6.64	77.22	1.68
Ilexsaponin A_1_	1	107.58	5.60	88.99	5.53
	50	105.76	3.96	86.25	7.21
	160	101.49	4.19	80.36	1.41
3β,19α-dihydroxyolean-12-ene-24, 28-dioic acid	20	96.50	6.20	78.44	1.83
	50	107.81	3.54	89.31	6.77
	160	99.29	1.22	75.44	8.91
Ilexgenin A	20	101.38	2.83	85.31	1.27
	50	106.04	2.44	90.41	7.83
	160	105.89	6.59	79.44	9.08

The linear regressions of IhD, IsA, ID and IA in the mice plasma exhibited good linear relationships over the range of 0.5–200, 0.5–200, 10–200 and 10–200 ng mL^−1^, respectively. The mean values of regression equation for IhD, IsA, ID and IA in the plasma were: *y* = 0.17448*x* + 0.32449 (*r* = 0.9989), *y* = 0.17175*x* + 2.27938 (*r* = 0.9969), *y* = 0.01351*x* + 0.01058 (*r* = 0.9980) and *y* = 0.02432*x* + 0.13488 (*r* = 0.9957), respectively.

The LLOQ of IhD, IsA, ID and IA were 0.5, 0.5, 10, 10 ng mL^−1^, respectively. The RSD and RE of the four analytes at the LLOQ were less than 14.35% and within±13.87%, respectively.

Intra-day accuracy and precision were determined by analyzing six replicates at three different concentration levels, six times at each concentration (Table 2). For all the samples evaluated, both RSD and RE were less than 15%.

**Table 2:** Precision and accuracy of the four analytes in mice plasma (*n* = 6)

Analytes	Spiked-concentration (ng mL^−1^)	Intra-run			Inter-run		
		Measured (ng mL^−1^)	RSD (%)	RE (%)	Measured (ng mL^−1^)	RSD (%)	RE (%)
Ilexhainanoside D	1	1.04 ± 0.12	11.79	3.86	1.02 ± 0.11	10.69	2.24
	50	47.83 ± 1.50	3.13	−4.33	48.26 ± 4.07	8.42	1.93
	160	148.94 ± 2.74	1.84	−6.91	163.51 ± 12.42	7.60	2.19
Ilexsaponin A_1_	1	1.05 ± 0.12	11.26	4.68	1.06 ± 0.10	9.47	6.11
	50	46.27 ± 2.83	6.11	−7.45	48.74 ± 3.81	7.81	1.95
	160	143.95 ± 1.47	1.02	−10.03	162.85 ± 14.56	8.94	1.78
3β,19α-Dihydroxyolean-12-ene-24, 28-dioic acid	20	18.04 ± 1.29	7.17	−9.81	19.34 ± 1.77	9.17	−3.29
	50	49.13 ± 2.95	6.00	−1.75	50.30 ± 3.86	7.67	2.01
	160	162.94 ± 11.21	6.88	1.83	167.11 ± 14.97	8.96	4.44
Ilexgenin A	20	17.83 ± 1.77	9.94	−10.85	19.46 ± 1.87	9.62	−2.68
	50	47.19 ± 2.59	5.50	−5.62	49.06 ± 4.26	8.68	1.96
	160	158.38 ± 14.4	9.09	−1.01	162.45 ± 13.96	8.59	1.53

Freeze–thaw stability, short-term temperature stability, long term stability and post-preparative stability were tested. Stability data was summarized in Table 3 and indicated this new method for the simultaneous determination of IhD, IsA, ID and IA offered satisfactory stability.

**Table 3:** Stability of the four analytes in mice plasma (*n* = 3)

Analytes	Spiked-concentration (ng mL^−1^)	Three freeze–thaw cycle		−20 °C for 1 month		4 h at room temperature		Processed samples at 4 °C for 12 h	
		Measured (ng mL^−1^)	RSD (%)	Measured (ng mL^−1^)	RSD (%)	Measured (ng mL^−1^)	RSD (%)	Measured (ng mL^−1^)	RSD (%)
Ilexhainanoside D	1	1.01 ± 0.07	6.99	0.98 ± 0.07	6.78	1.04 ± 0.12	11.10	1.06 ± 0.11	10.75
	50	50.39 ± 2.09	4.15	50.22 ± 5.46	10.88	51.93 ± 5.59	10.76	48.95 ± 4.56	9.31
	160	149.02 ± 7.91	5.31	164.68 ± 7.83	4.75	161.08 ± 12.81	7.95	152.27 ± 8.78	5.77
Ilexsaponin A_1_	1	1.00 ± 0.11	11.51	1.44 ± 0.20	13.73	1.06 ± 0.1	9.47	1.05 ± 0.08	7.67
	50	51.07 ± 3.61	7.07	50.76 ± 6.92	13.63	49.28 ± 4.22	8.56	50.21 ± 2.04	4.07
	160	141.42 ± 5.11	3.62	162.22 ± 9.96	6.14	161.21 ± 14.7	9.12	141.68 ± 4.38	3.09
3*β*,19*α*-Dihydroxyolean-12-ene-24, 28-dioic acid	20	21.53 ± 0.36	1.70	19.51 ± 2.57	13.17	20.4 ± 1.87	9.15	17.9 ± 0.67	3.73
	50	48.60 ± 0.64	1.31	51.90 ± 5.82	11.21	55.69 ± 4.99	8.96	52.49 ± 3.05	5.81
	160	173.51 ± 9.26	5.34	163.92 ± 14.95	9.12	155.41 ± 5.55	3.57	154.65 ± 2.45	1.58
Ilexgenin A	20	21.99 ± 0.89	4.03	19.27 ± 2.55	13.23	21.15 ± 2.24	10.57	18.17 ± 0.77	4.26
	50	49.00 ± 3.49	7.12	52.30 ± 5.97	11.41	53.39 ± 4.19	7.85	50.59 ± 3.05	6.02
	160	178.85 ± 14.56	8.14	155.78 ± 15.13	9.72	156.18 ± 7.39	4.73	153.89 ± 1.2	0.78

### 3.4. Application

The validated analytical method was applied to pharmacokinetics after oral administration of the IhS to mice at a dose of 250 mg kg^−1^. The different pharmacokinetic behaviors of IhD, IsA, ID and IA in the control and NAFLD mice were investigated.

#### 3.4.1. Confirmation of the success of the NAFLD model

Feeding of high-fat diet was known to cause NAFLD *in vivo* characterized by the rapidly increase of TC and ALT levels in serum. It was widely used in experimental animals to model human NAFLD.^20^

**Fig. 4:**
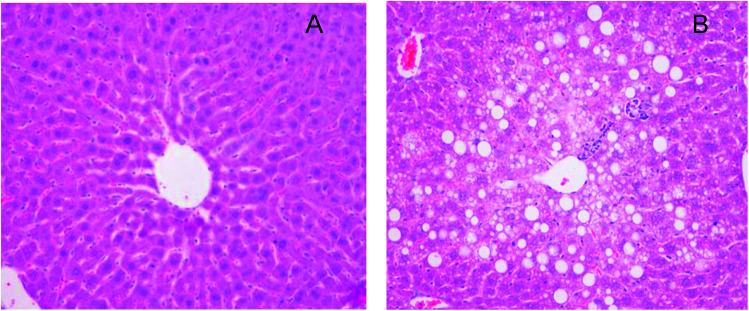
H&E stained cross-sections of representative liver tissues (magnification 200×). (A) The control mice (B) the NAFLD mice.

As previously described,^21–23^ in the model of NAFLD high-fat diet feeding induced body weight and liver weight, increased epididymal and perirenal fat accumulation (Table 4). Moreover, mice fed with high-fat diet showed significantly higher plasma total cholesterol and AST, ALT levels in comparison with control mice (+58%, +465%, +83%, respectively, *vs.* control) (Table 4). In addition, hepatic histopathological examination revealed numerous lipid droplets and steatosis in the NAFLD mice (Fig. 4). Based on the above results, the NAFLD model was considered to be successfully established.

**Table 4:** Biochemical characteristics of the control mice and the NAFLD mice (*n* = 6)^a^

Parameters	Unit	Control mice	NAFLD mice
Body weight	g	28.00 ± 1.99	45.24 ± 2.99**
Liver weight	g	1.02 ± 0.12	1.45 ± 0.21**
Epididymal fat	g	0.45 ± 0.10	2.38 ± 0.33**
Perirenal fat	g	0.11 ± 0.02	1.02 ± 0.11**
TG	mmol L^−1^	0.70 ± 0.15	0.87 ± 0.17
TC	mmol L^−1^	2.29 ± 0.25	3.62 ± 0.35**
LDL-c	mmol L^−1^	0.29 ± 0.07	0.57 ± 0.11**
HDL-c	mmol L^−1^	2.25 ± 0.25	3.77 ± 0.34
ALT	U/L	16.19 ± 4.56	91.43 ± 35.37**
AST	U/L	48.70 ± 8.39	84.34 ± 20.05**

a * *P* < 0.05 *vs.* control group.

#### 3.4.2. Pharmacokinetic study in control and NAFLD mice

**Fig. 5:**
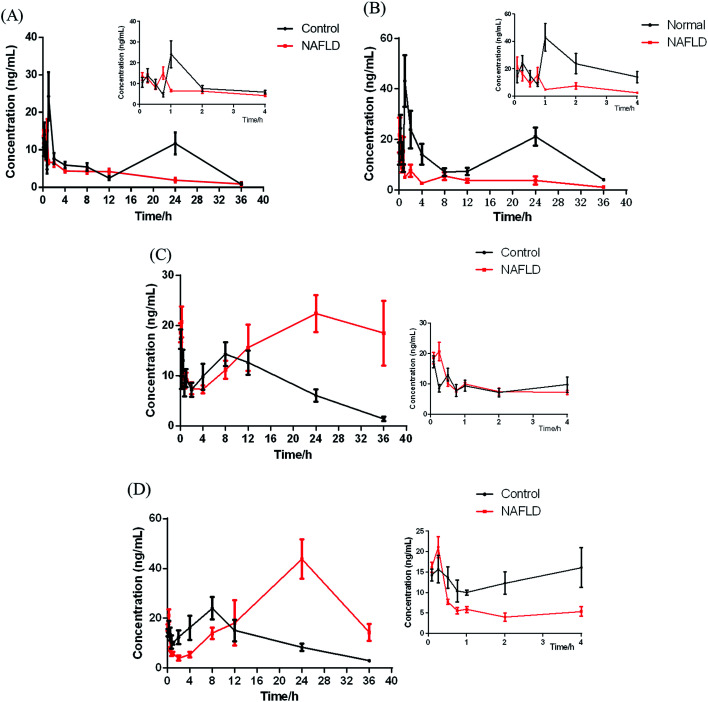
Mean plasma concentration–time curves for IhD (A), IsA (B), ID (C) and IA (D) in the control and NAFLD mice after oral administration of IhS. The smaller plots were the enlarged curves for 0–4 h. (*n* = 6).

The developed method was successfully applied in a pharmacokinetic study of IhD and IsA in C57BL/6J mice after oral administration of IhS. The mean plasma concentration–time profiles of IhD, IsA and their metabolites ID, IA are shown in Fig. 5. The main PK parameters were listed in Table 5.

**Table 5:** Pharmacokinetic parameters of IhD, IsA, ID and IA after oral administration of IhS (*n* = 6)^a^

Analytes	Group	Pharmacokinetic parameters							
		AUC_(0−t)_ (ng mL^−1^ × h)	AUC_(0−∞)_ (ng mL^−1^ × h)	MRT_(0−t)_ (h)	*t*_1/2_ *z* (h)	*T*_max_ (h)	*C*_max_ (ng mL^−1^)	*V*_d_ (mL g^−1^)	CL*z*/F (mL h^−1^/g)
Ilexhainanoside D	Control	239.90	259.64	17.02	16.42	1	24.27	8785.14	370.88
	NAFLD	105.70	125.43	10.66	11.98	0.75	15.15	14 992.90	867.21
Ilexsaponin A_1_	Control	469.37	653.13	17.38	31.11	1	37.25	8139.84	181.37
	NAFLD	133.86	150.60	13.62	13.34	0.083	21.78	16 745.73	869.81
3*β*,19*α*-Dihydroxyolean-12-ene-24, 28-dioic acid	Control	300.24	317.77	12.97	8.48	0.083	17.33	3961.81	323.95
	NAFLD	584.92	—	19.48	—	24	22.42	—	—
Ilexgenin A	Control	421.13	464.70	13.08	10.22	8	24.02	4283.20	290.53
	NAFLD	942.25	—	20.28	—	24	43.79	—	—

a **P < 0.01 *vs.* control group.

In this study, UPLC-MS/MS was used to increase sensitivity and decrease detection limit. Compared with the control group, a shorter *T*_max_ was observed for IhD and IsA in NAFLD group, which indicated that their absorption processes became faster. It was also found that *C*_max_, AUC_(0−*t*)_ and AUC_(0−∞)_ of IhD and IsA significantly decreased, and the *t*_1/2_ and MRT_(0−*t*)_ decreased in the NAFLD group by comparing with the control group.

The results showed that ID and IA could be detected in plasma after oral administration of IhS, which indicated that IhD and IsA were converted to their aglycones (ID and IA) *in vivo*. The AUC and *T*_max_ of the two aglycones increased greatly in the NAFLD group compared with the control group, which indicated that the absorptions of ID and IA in the NAFLD mice were promoted. The MRTs of ID and IA were increased in the NAFLD mice. The result was consistent with previous reference of a higher MRT of IA in the NAFLD rats.^10^ Meanwhile, the double peak phenomena were observed for the two analytes. These were normally occurred in the pharmacokinetics of traditional Chinese medicines.^24–26^ The result was similar to the pharmacokinetics study of triterpenoids from *Rhizoma alismatis*.^27^ These phenomena might be attributed to some factors that include enterohepatic recirculation, variable gastric emptying, multiple sites absorption, formulation *etc.* However, further research is needed to elucidate the mechanism of such double peak phenomena.

The total amount of the IhD, IsA, ID and IA absorbed in the NAFLD mice was similar to it in the control group. The proportion of saponins absorbed in the control group was greater than those in the NAFLD group. However, the aglycones absorbed in the NAFLD group were greater than those in the control group. The metabolites, especially IA was reported to treat NAFLD^8^ and was more easily absorbed with stronger biological activity. The study droped a hint that converting saponins into aglycones might be a way for this herb to improve NAFLD.

## 4. Conclusion

A rapid, sensitive and convenient UPLC-MS/MS method for simultaneous determination on two pairs of oleanene- and ursane-type triterpenoids (IhD and IsA, ID and IA) in mice plasma was developed, validated and successfully applied in a comparative pharmacokinetic study. For the biosample preparation of oleanene- and ursane-type isomeric triterpenoids, LLE was better than protein precipitation. Compared with changing second order MS (MS^2^) conditions, the adjustment of LC conditions was more effective for the separation of isomers. The pharmacokinetic behaviors of triterpenoids in NAFLD mice were different from them in control mice, which were in accordance with the previous reports.^10^

This is the first report on the determination of the major triterpenoid saponins from *I. hainanensis* and their major metabolites in biosamples. The simple method developed here and the pharmacokinetic parameters obtained would prove useful in clinical applications and further new drug development of *I. hainanensis*.

## Conflicts of interest

There are no conflicts to declare.

## Supplementary Material
